# Poly(ε-Caprolactone)/Sodium Bicarbonate/β-Tricalcium Phosphate Composites: Surface Characterization and Early Biological Response

**DOI:** 10.3390/ma18112600

**Published:** 2025-06-03

**Authors:** Alessandro Mosca Balma, Riccardo Pedraza, Clarissa Orrico, Sara Meinardi, Tullio Genova, Giovanna Gautier di Confiengo, Maria Giulia Faga, Ilaria Roato, Federico Mussano

**Affiliations:** 1 Bone and Dental Bioengineering Laboratory, CIR Dental School, Department of Surgical Sciences, University of Turin, 10126 Turin, Italy; alessandro.moscabalma@unito.it (A.M.B.); or riccardo.pedraza@stems.cnr.it (R.P.); or clarissa.orrico@polito.it (C.O.); ilaria.roato@unito.it (I.R.); 2 Department of Mechanical and Aerospace Engineering, Politecnico di Torino, Corso Duca degli Abruzzi 24, 10129 Turin, Italy; 3 Institute of Sciences and Technologies for Sustainable Energy and Mobility, National Council of Research, Strada delle Cacce 73, 10135 Turin, Italy; giovanna.gautier@stems.cnr.it (G.G.d.C.); mariagiulia.faga@stems.cnr.it (M.G.F.); 4 Department of Life Sciences and Systems Biology, University of Torino, Via Accademia Albertina 13, 10123 Torino, Italy; sara.meinardi@edu.unito.it (S.M.); tullio.genova@unito.it (T.G.)

**Keywords:** poly-ε-caprolactone (PCL), β-tricalcium phosphate (β-TCP), sodium bicarbonate, bone substitute material, adipose stem cells, cellular adhesion, cell viability, nanoindentation, pH

## Abstract

Bone graft substitutes combining the mechanical features of poly-ε-caprolactone (PCL) and the bioactivity of β-tricalcium phosphate (β-TCP) have been widely reported in the literature. Surprisingly, however, very little is known about the incorporation of carbonate at a biomimicking level. The authors studied β-TCP/PCL composites at 20 wt.% and 40 wt.%, either enriched or not with sodium bicarbonate (at 2 wt.% and 4 wt.%), through SEM and EDX analyses; surface free energy estimation; pH measurement after 1, 2, and 3 days of incubation in cell media; nanoindentation; and a protein adsorption test with bovine serum albumin. The early biological response was assessed using adipose mesenchymal stem cells, as an established in vitro model, via cellular adhesion (20 min), spreading (24 h), and viability assays (1, 3, 7 days). By increasing the β-TCP content, the composites’ hardnesses and Young’s moduli (EiT) were improved, as well as their protein adsorption compared to neat PCL. Sodium bicarbonate increased the polar component of the surface energy, alkalinized the composite with a higher β-TCP content, and attenuated its early negative cell response. Further investigation is needed to deepen the knowledge of the mechanisms underpinning the mechanical features and long-term biological behavior.

## 1. Introduction

Bone healing capacity, although intrinsically high, can be challenged by non-union fractures, oncologic resections, craniofacial malformations, resulting in large bone defects with long-term disability and pain [1]. To increase bone volume, where insufficient, surgeons perform bone repair procedures based on tissue grafts (autografts, allografts, xenografts) and artificial scaffolds (alloplasts) [2]. The cumulative economic burden thereof was estimated to be USD 17 billion per year [2].

Regarded clinically as the gold standard until recently, autografts, even though both osteoinductive and osteoconductive, are hindered by donor-site morbidity and the scarcity of available bone [3,4]. Allografts and xenografts may be prone to immune rejection by the recipient and disease transmission, and finally the result is less osteoinductive than autografts, owing to an often poorly conservative manufacturing process [4]. Propelled by advances in regenerative medicine, artificial scaffolds [3] hold great promise, but achieve low fusion rates, mainly due to limited cell ingrowth, and induce local aseptic inflammation upon degradation [4,5].

More research is therefore needed to improve the performance of artificial scaffolds, improving their biological response. Calcium phosphate-based ceramics (CaPs), i.e., hydroxyapatite (HA) and β-tricalcium phosphate (β-TCP), are the most obvious choice as alloplasts, in terms of biomimicry, since HA is the primary mineral phase of bone, accounting for 65% of its weight [6]. While all CaPs are osteoconductive [7], only specific types are osteoinductive [8]. β-TCP is resorbable [9], osteoinductive [10], and similar to the bone mineral phase, with equivalent rates of clinical success compared to autografts according to some authors [11].

However, CaPs’ favorable mechanical features, such as structural strength and compression resistance, do not overcome their fragility and low stiffness [12]. Hence, a viable approach could entail the combination of CaPs and resorbable polymers. Several polymers that have been proposed as bone substitutes are endowed with good flexibility, low weight and ductility, and controllable degradation [13], but show low rigidity [14]. Among them, poly-ε-caprolactone (PCL) is one of the most commonly used thermoplastic polyesters [15]. At physiological temperature, PCL has a low tensile strength and high elasticity, due to the presence of amorphous regions in its semicrystalline lattice [16]. Owing to a relatively low melting temperature (around 60 °C), PCL is easily printable [17]. As its complete dissolution may require up to 2–3 years [18], it is indicated for long-term applications in bone tissue replacement, where good mechanical properties and prolonged stability over time are sought [19].

Bone graft substitutes combining the mechanical features of poly-ε-caprolactone and the bioactivity of β-TCP [20] have already emerged as a viable option. Challenges remain, however, in replicating both the complex multiscale hierarchy of bone structure and the sophisticated chemical composition of bone components. Surprisingly, to the best of our knowledge, no research has focused on the fact that, for truly replicating the bone matrix chemical composition, β-TCP/PCL should also incorporate low levels of carbonates [21]. Yet, according to a famous textbook in the bone field [21], “bone mineral is known to be composed of highly substituted, poorly crystalline carbonated apatite”, in which “the substitution of carbonate ions into phosphate sites, can occupy up to 6% of the total mineral by weight”. Staring from a careful biomimicry, therefore, the authors aimed at comparing the early biological response of different β-TCP/PCL composites, either enriched or not with sodium bicarbonate at physiological concentrations, as bone substitute materials.

## 2. Materials and Methods

### 2.1. Sample Preparation

Polycaprolactone matrix (CELLINK PCL TP-60505, Bico Group, Gothenburg, Sweden) was charged with two concentrations 20 wt.% (PCL/β-TCP 80/20) and 40 wt.% (PCL/β-TCP 60/40) of β-tricalcium phosphate (CAS-No.: 7758-87-4; Sigma-Aldrich, St. Louis, MO, USA) through solvent casting with chloroform (CHCl_3_, CARLO ERBA Reagents s.r.l., Cornaredo, Italy), as previously described [22]. Another two composites were prepared by substituting 10% of the filler quantity in PCL/β-TCP 80/20 and PCL/β-TCP 60/40 with sodium bicarbonate (CAS No: 144-55-8; Merck Life Science S.r.l., Milano, Italy), obtaining, respectively, samples with 18 wt.% β-TCP/2 wt.% sodium bicarbonate (PCL/β-TCP/NaHCO_3_ 80/18/2) and 36 wt.% β-TCP/4 wt.% sodium bicarbonate (PCL/β-TCP/NaHCO_3_ 60/36/4). In summary, along with neat PCL, which was kept as a control, four different materials were prepared: PCL/β-TCP 80/20, PCL/β-TCP 60/40, PCL/β-TCP/NaHCO_3_ 80/18/2, and PCL/β-TCP/NaHCO_3_ 60/36/4 (Table 1).

### 2.2. SEM and EDX Analyses

A scanning electron microscope (Phenom XL G2 Desktop SEM, Thermo Fisher Scientific, Waltham, MA, USA) (SEM) was employed to study the microstructure of the samples. Specimens were washed in distilled water, rinsed thoroughly in a 70-30 ethanol–water solution, polished ultrasonically in 99.9% ethanol for 20 min, and after a drying process under a chemical hood, coated with a ~10 nm conductive layer of gold. The SEM parameters adopted for acquisition and analyses were 10 kV of voltage V in the MAP configuration with a back-scatter detector (BSD). The correct filler dispersion was evaluated with EDX analyses carried out on the surface of the composites.

### 2.3. Contact Angle and Surface Free Energy

Contact angles were measured with a Biolin Scientific Theta Lite optical tensiometer (Stockholm, Sweden) using the sessile drop method, with double-distilled water (dH_2_O) and diiodomethane (CH_2_I_2_, CAS-No.: 75-11-6, Sigma-Aldrich, St. Louis, MO, USA), as described elsewhere [22]. The Owens–Wendt–Rabel–Kaelbele (OWRK) technique was used to compute the surface free energy [22]. Simple linear regression was used to derive total (γ), polar (γP), and dispersive (γD) components, with dH_2_O and CH_2_I_2_ values serving as standard constants (Table 2).

### 2.4. Nanoindentation

Nanoindentation tests were performed using a FISCHERSCOPE HM 2000 XYm (Helmut Fischer GmbH, Sindelfingen, Germany) fitted with a Vickers indenter. An acrylic plate reference sample was used to calibrate the indenter tip before testing, allowing accurate definition of the predicted contact area (A_C_) from the contact penetration depth (h_C_). Ten indentations were performed in a load-control mode up to 3 mN. A load–hold–unload cycle was used, with a 20 s loading period, a 5 s holding period, and a 20 s unloading period. Load–depth curves, hardness values, and the reduced modulus were evaluated. The translation stage was moved to choose random test sites under an optical microscope. Nanoindentation tests were performed on cylindrical flat samples of 8 mm in diameter and 3 mm in thickness.

### 2.5. pH Evaluation

The pH was evaluated in cell culture medium (Alpha-MEM Life Technologies, Milan, Italy) using a test tube LLG-pH Pen (Am Hambuch 1-3, 53340 Meckenheim, Germany). To reproduce as closely as possible the conditions of the cellular tests, the same volume of medium (200 µL) per sample was used. The pH evaluation was carried out at 3 time points: 1, 2, and 3 days.

### 2.6. Protein Adsorption

A protein adsorption assay was performed by covering the samples with a 5% solution of bovine serum albumin (BSA CAS-No.: 9048-46-8, Merck Group, Darmstadt, Germany) in phosphate-buffered saline (PBS, Euroclone, Pero, Italy). After incubation at 37 °C for 20 min, specimens were washed twice with PBS. According to the manufacturer’s instructions, the result was subsequently quantified through a Pierce™ BCA Protein Assay Kit (Life Technologies, Carlsbad, CA, USA).

### 2.7. Cellular Tests

Adipose stem cells ASC52hTert (ATCC, Manassas, VA, USA) were cultured in Alpha-MEM (Life Technologies, Milan, Italy) supplemented with 10% fetal bovine serum (FBS, Euroclone, Pero, Italy), 100 U/mL penicillin, and 100 μg/mL streptomycin (Life Technologies, Carlsbad, CA, USA) to study the biological response in vitro. To prevent contact inhibition, cells were passed at sub-confluence and maintained at 37 °C in a humidified environment containing 5% CO_2_.

#### 2.7.1. Cell Adhesion and Cell Spreading

Cells were seeded at 7000 cells/well on 96-well plate samples with different percentages of filler. A cell adhesion analysis was performed by plating ASCs for 20 min, then fixing in 4% paraformaldehyde solution, and washing with PBS, then cell nuclei were stained by 1 μM DAPI (CAS-No.: 28718-90-3, MBD0020 Sigma-Aldrich, St. Louis, MO, USA) for 15′ at 37 °C.

To evaluate cellular morphology, cells were maintained in culture for 24 h, then they were fixed and stained with both phalloidin (Alexa Fluor^®^ 488 Phalloidin, Cell Signaling technology, Danvers, MA, USA) for the cytoskeleton and DAPI for cell nuclei. Immunofluorescence microscopy images were acquired using a Nikon Eclipse Ti-E microscope with Nikon Plan 40×/0.75 and Nikon Plan 10×/0.10 objectives (Nikon Corporation, Tokyo, Japan). Cell nuclei were counted using the ‘analyze particles’ tool of the ImageJ software (Version 2.14.0/1.54f, ImageJ, U. S. National Institutes of Health, Bethesda, MD, USA). For cellular morphology analysis, four different fields were acquired at a “higher magnification” on each of the three samples for each composite, then processed with the cellpose [23,24] cyto3 segmentation algorithm, and pre-trained with other similar images in a controlled learning process to obtain the contours of the single cell on all the images. Twelve different shape descriptors were measured for each detected cell with the Set Measurements function implemented in Fiji/ImageJ (i.e., area, perimeter, best fitting ellipse (BFE) major axis, BFE minor axis, BFE aspect ratio, BFE angle, circularity, roundness, solidity, Feret’s diameter, Feret’s angle, and minimum caliper diameter). Six out of these twelve parameters were selected as relevant data for cell spreading evaluation (area, perimeter, BFE aspect ratio, BFE angle, circularity, and roundness). A MATLAB (MATLAB R2024a; The MathWorks, Inc., Natick, MA, USA) script was implemented and used to analyze and plot the data.

#### 2.7.2. Cell Viability

ASCs were seeded onto various specimens at a density of 10,000 cells per well in 96-well culture plates, in alpha-MEM with 10% FBS and 1% penicillin/streptomycin. The viability evaluation was performed after 1, 3, and 7 days of in vitro culture using the Cell Titer GLO kit (Promega, Milan, Italy), following the manufacturer’s instructions. The results were measured in relative light units (RLUs).

### 2.8. Statistical Analysis

The statistical analyses were carried out using the Stata software (version 18.0; StataCorp, College Station, TX, USA). A one-way ANOVA was used to compare differences in group variances at different time steps, and a Bonferroni post hoc correction coefficient was used to determine which groups varied statistically significantly. Repeated t-tests were employed to conduct a statistical analysis on shape descriptors to assess cell morphology. An alpha significance level of 0.05 was used [25].

## 3. Results

### 3.1. SEM and EDX Analyses

**Figure 1:**
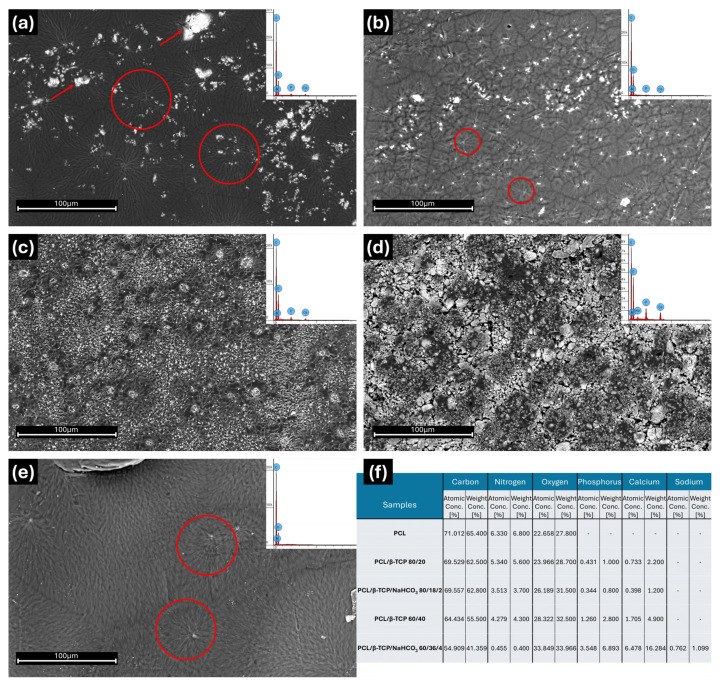
Scanning electron micrographs of samples, performed at a 1500× magnification, and EDX spectra (top right corner of the images): (**a**) PCL/β-TCP 80/20, (**b**) PCL/β-TCP/NaHCO_3_ 80/18/2, (**c**) PCL/β-TCP 60/40, (**d**) PCL/β-TCP/NaHCO_3_ 60/36/4, and (**e**) neat PCL. (**f**) Table of elements detected on samples’ surfaces with EDX (red circles highlight clusters of PCL stripes; red arrows point out filler aggregates).

Micrographs representing the surface of the samples are reported in Figure 1. Neat PCL, PCL/β-TCP 80/20, and PCL/β-TCP/NaHCO_3_ 80/18/2 (Figure 1a–c) shared a clear similarity of their surface morphology, characterized by random clusters of PCL stripes (red circles in Figure 1a,b,e), most likely due to solvent evaporation during the casting process. This characteristic was not highlighted on the composites at a higher concentration of β-TCP, particularly on the sample containing sodium bicarbonate (PCL/β-TCP/NaHCO_3_ 60/36/4). Indeed, the presence of NaHCO_3_ seemed to improve β-TCP dispersion inside the PCL matrix and to limit the formation of filler aggregates during the drying process. A better filler dispersion, when comparing PCL/β-TCP 80/20 with PCL/β-TCP/NaHCO_3_ 80/18/2 and PCL/β-TCP 60/40 with PCL/β-TCP/NaHCO_3_ 60/36/4, was observed in the former case, in the reduced dimensions of the filler aggregates (red arrows in Figure 1a). In the latter case, there was a clear decrease in the number of clusters formed during solvent evaporation, and a consequent higher homogeneity of the composite PCL/β-TCP/NaHCO_3_ 60/36/4.

According to the EDX analyses performed on each surface (Figure 1f), carbon was the most represented element, reaching the highest value in neat PCL and the lowest in PCL/β-TCP/NaHCO_3_ 60/36/4. On the contrary, oxygen was the highest in PCL/β-TCP/NaHCO_3_ 60/36/4 and the lowest in neat PCL; it increased with the β-TCP and NaHCO_3_ contents. Nitrogen was inversely correlated with the filler quantity and was reduced in the presence of bicarbonate. Notably, PCL/β-TCP 80/20 and PCL/β-TCP 60/40 differed from their NaHCO_3_-containing counterparts, and PCL/β-TCP/NaHCO_3_ 60/36/4 presented by far the lowest values of nitrogen. As expected, phosphorus was not detectable in neat PCL, while it progressively increased in PCL/β-TCP 80/20 and PCL/β-TCP 60/40; it was also less detected in PCL/β-TCP/NaHCO_3_ 80/18/2 than in PCL/β-TCP 80/20, but, remarkably, it was present in double the amount in PCL/β-TCP/NaHCO_3_ 60/36/4 compared to PCL/β-TCP 60/40. Calcium reproduced a similar pattern, with a 3.5-fold increase in PCL/β-TCP/NaHCO_3_ 60/36/4 compared to PCL/β-TCP 60/40. Finally, small amounts of sodium could be measured only in PCL/β-TCP/NaHCO_3_ 60/36/4.

### 3.2. Contact Angle and Surface Free Energy

**Figure 2:**
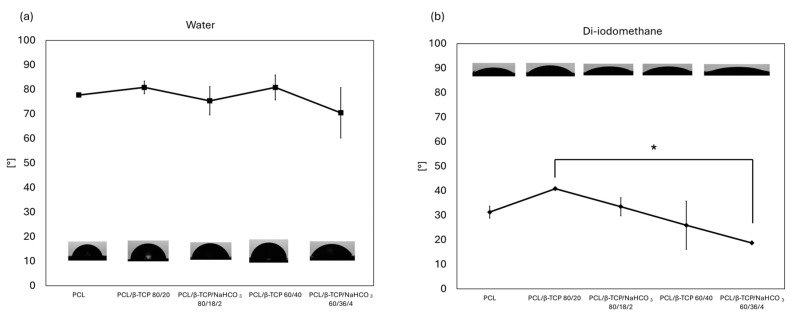
Image representing OCA values of specimen in (**a**) hydrophilic and (**b**) lipophilic environment (* for *p* < 0.05).

The hydrophilic and the lipophilic behaviors of the samples are shown in Figure 2. Regarding the hydrophilic environment, applying the polar solvent (water) optical contact angle (OCA) did not show any relevant statistical difference among samples, settling in a range between ~70° and ~80°. These results suggest a common behavior in an aqueous environment. On the contrary, in the lipophilic context, the materials behaved differently. All OCAs were lower than in a hydrophilic environment, revealing a marked affinity with the apolar solvent (diiodomethane). PCL/β-TCP/NaHCO_3_ 80/18/2 reached the highest value among the samples and PCL/β-TCP/NaHCO_3_ 60/36/4 was the lowest; in general, the higher the amount of filler, the higher the affinity with the apolar solvent. Comparing samples with and without NaHCO_3_, in both cases the OCA followed the same trend: specimens with sodium bicarbonate appeared slightly more lipophilic than their counterparts. A statistically significant difference (*p* < 0.05), however, was found only between PCL/β-TCP 80/20 and PCL/β-TCP/NaHCO_3_ 60/36/4.

Table 3 reports the values of surface free energy (SFE) calculated through the OWRK method. The total surface energy (γ) was the lowest for PCL/β-TCP 80/20 and the highest for PCL/β-TCP/NaHCO_3_ 60/36/4. Adding sodium bicarbonate increased γ, i.e., PCL/β-TCP/NaHCO_3_ 80/18/2 and PCL/β-TCP/NaHCO_3_ 60/36/4 exhibited, respectively, higher values than PCL/β-TCP 80/20 and PCL/β-TCP 60/40. This was mainly due to the variation in the dispersive component in the 20 wt.% compounds and to the variation in the polar component in the 40 wt.% specimens.

### 3.3. Nanoindentation

**Figure 3:**
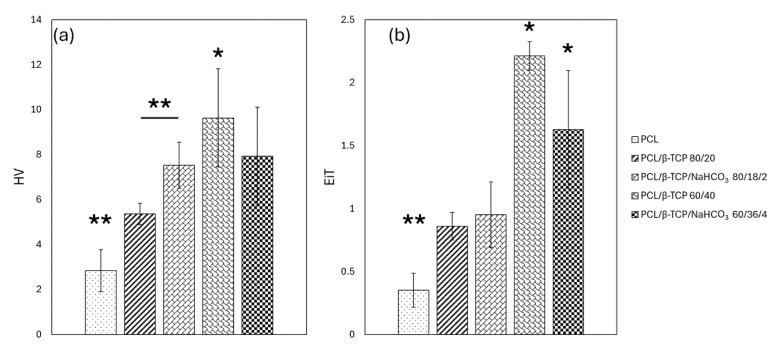
Graphs representing HV (**a**) and EiT (**b**) results ((*) for *p* < 0.05 and (**) for *p* < 0.01).

The indentation data (Figure 3) revealed that the Vickers hardness (HV) and Young’s moduli (EiT) values increased when increasing the β-TCP content, probably due to the strength enhancement effect of filler inside the polymer matrix. The rise, compared to neat PCL, is particularly evident from 20 to 40 wt.%. The addition of NaHCO_3_ in the PCL/β-TCP composites resulted in a relevant decrease in both HV and EiT values in specimens with 40 wt.% total filler content. On the contrary, the sodium bicarbonate significantly enhanced the hardness of PCL/β-TCP 80/20 (*p* << 0.01), while it did not alter the mechanical properties. Indeed, the increase in hardness and Young’s modulus compared with the pure polymer is evident. However, the differences between samples containing ~20% and ~40% β-TCP appeared less pronounced in the presence of NaHCO_3_, especially in the case of hardness, where the average values could not be considered significantly different.

### 3.4. pH

**Figure 4:**
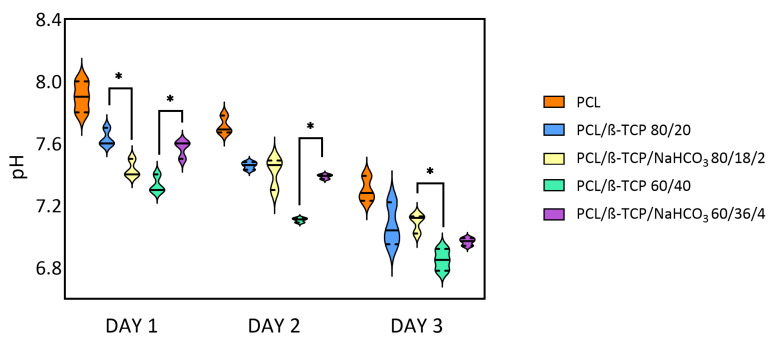
pH measurement at day 1, day 2, and day 3 ((*) for *p* < 0.05).

The pH measurements after keeping the samples for 1, 2, and 3 days in cell culture medium are reported in Figure 4. Overall, the pH of alpha-MEM tended to decrease, always remaining around pH 7 owing to the medium’s buffer effect. At all time steps, neat PCL differed statistically from the other compounds, showing a more basic behavior. In the formulation containing 4 wt.% sodium bicarbonate, an alkalizing buffering effect occurred, with statistical significance, comparing PCL/β-TCP 60/40 with PCL/β-TCP/NaHCO_3_ 60/36/4 at 1 and 2 days. An initially opposite statistically significant (*p* < 0.05) effect (acidification) was evident at 24 h for PCL/β-TCP 80/20 and PCL/β-TCP/NaHCO_3_ 80/18/2, which was attenuated at the subsequent time steps, with the two conditions reaching similar pH levels with no significant differences.

### 3.5. Protein Adsorption

**Figure 5:**
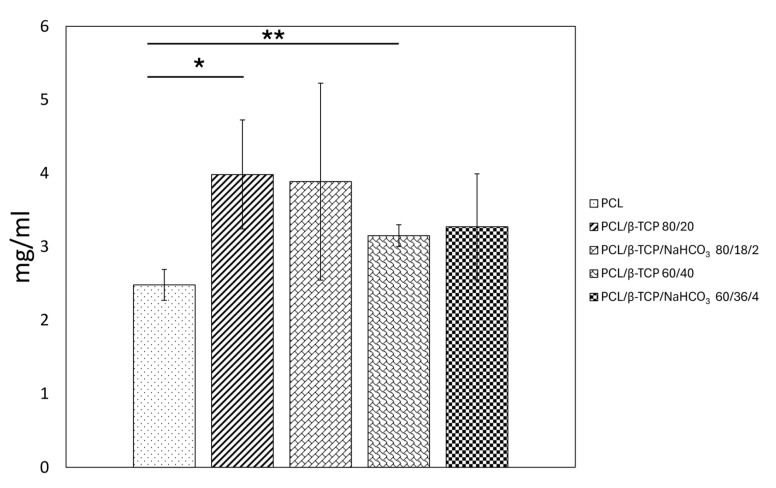
Graph showing the total amount of BSA protein adsorbed on each sample ((*) for *p* < 0.05 and (**) for *p* < 0.01).

The amount of BSA protein adsorbed on the sample surfaces was higher for compounds with β-TCP (Figure 5). Neat PCL displayed the lowest value (~2.5 mg/mL), differing in a statistically significant way (*p* = 0.032) from PCL/β-TCP 80/20, which adsorbed the highest amount of protein (~4 mg/mL) out of all the tested conditions. Also, PCL/β-TCP 60/40 adsorbed a higher amount of protein compared to PCL (*p* = 0.007). No relevant differences were found between either PCL/β-TCP 80/20 and PCL/β-TCP/NaHCO_3_ 80/18/2 or between PCL/β-TCP 60/40 and PCL/β-TCP/NaHCO_3_ 60/36/4 (*p* > 0.05).

### 3.6. Cellular Tests

#### 3.6.1. Cell Adhesion and Cell Spreading

**Figure 6:**
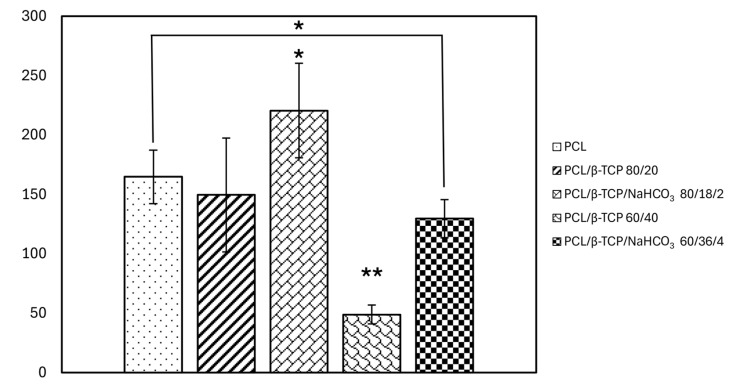
Graph representing cell adhesion assay, performed on samples after 20 min of culture in incubator ((*) for *p* < 0.05 and (**) for *p* < 0.01).

The number of adherent cells after 20 min (Figure 6) was significantly the highest for PCL/β-TCP/NaHCO_3_ 80/18/2, compared to all the other specimens. Considering the samples with an equal amount of filler, an increase in terms of cellular adhesion is noticeable when PCL/β-TCP 80/20 and PCL/β-TCP 60/40 are compared to their respective counterparts containing bicarbonate (*p* = 0.032 and *p* = 0.001). PCL/β-TCP 60/40 was, indeed, the sample on which the greatest decrease in the number of adherent cells occurred overall. However, except for PCL/β-TCP/NaHCO_3_ 80/18/2, no composites outperformed PCL in a statistically significant way.

**Figure 7:**
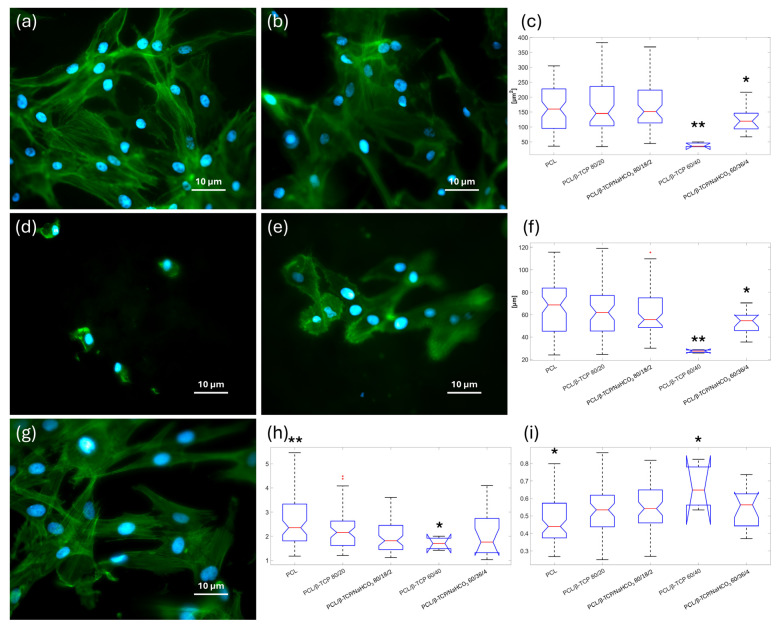
Images of immunofluorescence microscopy performed on ASCs plated on (**a**) PCL/β-TCP 80/20, (**b**) PCL/β-TCP/NaHCO_3_ 80/18/2, (**d**) PCL/β-TCP 60/40, (**e**) PCL/β-TCP/NaHCO_3_ 60/36/4, and (**g**) PCL after 24 h of incubation (nuclei of cells represented in blue; cytoskeletons stained in green). The other graphs represent the (**c**) area, (**f**) perimeter, (**h**) aspect ratio, and (**i**) circularity shape descriptor values ((*) for *p* < 0.05 and (**) for *p* < 0.01).

The shape descriptor of the BFE angle, calculated on the cell segmented from the spreading analysis (Figure 7a,b,d,e,g), did not show any preferential orientation of ASCs along a particular direction on any of the solvent-cast smooth substrates. Area and perimeter (Figure 7c,f) values were not significantly different among the samples, except for PCL/β-TCP 60/40 and PCL/β-TCP/NaHCO_3_ 60/36/4, where the cells were smaller in terms of dimensions and less rich in filopodia structures, a feature of well-spread cells. These two parameters were slightly smaller for PCL/β-TCP 80/20 and PCL/β-TCP/NaHCO_3_ 80/18/2 cells than for those grown on neat PCL; however, not in a significant way. Consistent with the perimeter parameter, the aspect ratio (Figure 7h) values were significantly higher for the neat polymer compared to the composites. The PCL/β-TCP 60/40 specimens showed the lowest value in terms of aspect ratio (Figure 7h) owing to the more rounded ASCs; this was also in accordance with the complex descriptors of circularity (Figure 7i) and roundness, where the resulting values were near to 1. These last two variables showed a statistically significant difference between neat PCL and all the other specimens (*p* < 0.05). Furthermore, the composites with the same amount of filler only varied from their bicarbonate-containing counterparts in a statistically relevant way at higher concentration of β-TCP. Specifically, sodium bicarbonate improved the properties of the cells adherent to PCL/β-TCP 60/40 in terms of mean area (*p* << 0.01), perimeter (*p* << 0.01), aspect ratio (*p* = 0.043), and circularity (*p* = 0.036).

#### 3.6.2. Viability

**Figure 8:**
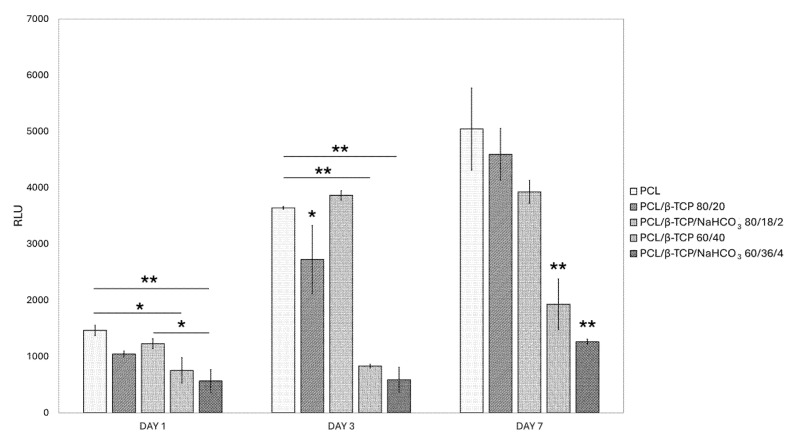
Viability results in relative light units (RLUs) at days 1, 3, and 7 ((*) for *p* < 0.05 and (**) for *p* < 0.01).

A cell viability analysis shows that all the samples proved to be biocompatible, since they were capable of sustaining cell proliferation, even though with different growth rates (Figure 8). Neat PCL always outperformed the 40 wt.% composites, being slightly surpassed, although not in a statistically significant way, only by PCL/β-TCP/NaHCO_3_ 80/18/2, at day 3. Moreover, albeit reaching minor RLU values, PCL/β-TCP 80/20 and PCL/β-TCP/NaHCO_3_ 80/18/2 did not differ significantly from neat PCL at day 7. At each time point, PCL/β-TCP 60/40 and PCL/β-TCP/NaHCO_3_ 60/36/4 showed a statistically significantly (*p* < 0.01) lower proliferation rate compared to the other composites.

## 4. Discussion

The purpose of this work was to evaluate the early biological response elicited by different β-TCP/PCL composites, with the specific aim of assessing whether the addition of sodium bicarbonate could be favorable. The printable polymer PCL was blended with the widely used calcium phosphate-based ceramic β-TCP, obtaining two formulations, namely, PCL/β-TCP 80/20 and PCL/β-TCP 60/40, to which two counterparts enriched with sodium bicarbonate were added (PCL/β-TCP/NaHCO_3_ 80/18/2 and PCL/β-TCP/NaHCO_3_ 60/36/4). Sodium bicarbonate has been used for porogenesis in conjunction with citric acid (or other acids) in the preparation of bone cements, with good results [26,27,28]. Notably, the functional importance of bicarbonate gained interest at a clinical level, as a decreased carbonate/phosphate ratio in cortical and cancellous bone may be a significant explanatory variable for fracture [29]. Also, as reported above, carbonate ions substituted into phosphate sites can occupy up to 6% of the total mineral bone by weight [21]. Hence, the authors wished to test whether biomimicking the bone mineral composition more closely in alloplastic scaffolds for bone substitution could have beneficial effects in terms of early cell response [30]. To the authors’ knowledge, this is the first time that sodium bicarbonate has been employed structurally, owing to its physiologic presence.

SEM micrographs highlighted the formation of residual random “star-like” PCL clusters, likely resulting from solvent evaporation during the casting process, that were dimensionally reduced with an increase in the β-TCP content, especially in the presence of NaHCO_3_. A possible explanation for this phenomenon relies on the fact that, since only the polymeric phase is completely soluble in chloroform, by increasing β-TCP, the available PCL surface may have been reduced during the drying process. Furthermore, by substituting one tenth of the β-TCP with NaHCO_3_, the total filler dispersion inside the compounds was improved, suggesting the positive role of sodium bicarbonate as a dispersing agent, possibly counteracting, at least partially, the van der Waals forces that could lead to particle aggregation in the presence of high amounts of filler [31].

This seems to be in accordance with the EDX analysis, which highlighted that phosphorus and calcium, respectively, more than doubled (atomic concentration % 3.5 vs. 1.3) and increased three-fold (atomic concentration % 6.5 vs. 1.7) on the surface of PCL/β-TCP/NaHCO_3_ 60/36/4 compared to PCL/β-TCP 60/40, which must depend on the surface distribution of the filler, since paradoxically the PCL/β-TCP/NaHCO_3_ 60/36/4 is less rich in P and Ca than PCL/β-TCP 60/40. Likewise, nitrogen was inversely correlated with the filler quantity and was reduced in the presence of bicarbonate. A mechanistic explanation regarding this peculiar distribution of the chemical elements available on the surface clearly correlated to NaHCO_3_ deserves thorough examination and will be the object of future experiments.

It is also noteworthy that sodium bicarbonate increased the polar component of the surface free energy of the samples, while the dispersive component (γ^D^) was higher in the 40 wt.% samples compared to the other composites and neat PCL. The only statistically significant difference concerning the OCA of diiodomethane, however, was found between PCL/β-TCP 80/20 and PCL/β-TCP/NaHCO_3_ 60/36/4, which had the highest total SFE. The preferential lipophilic features of the composites tested here are consistent with the behavior reported for several polymers [32], and the high level of surface free energy displayed by β-TCP is in accordance with Gao [33].

As known from Refs. [34,35], incorporating a filler within a polymeric matrix may improve the mechanical properties of the neat material, working as a toughening phase in the composites. This was also confirmed here, where the percentage increase of β-TCP filler within the PCL improved samples’ hardnesses (HV) and EiT Young’s moduli (EiT). Sodium bicarbonate significantly increased the hardness of PCL/β-TCP/NaHCO_3_ 80/18/2 compared to PCL/β-TCP 80/20, while maintaining its elastic properties. However, that was not the case for PCL/β-TCP/NaHCO_3_ 60/36/4, whose mechanical features became inferior, albeit not in a statistically significant way, to those of PCL/β-TCP 60/40. As stated above, the effect of NaHCO_3_ on the dispersion of β-TCP within the polymer is likely to play a role at the interface between filler particles and PCL, according to its reported porogenic activity [26,27,28].

Neat PCL was more basic than the composites, which is consistent with Juan et al. [36], who monitored the pH of different PCL/β-TCP formulations for 15 weeks. Indeed, the theoretical acidic behavior of PCL, owing to the release of carboxylates, is limited by its very slow degradation kinetics [13]. The expected alkalizing effect of sodium bicarbonate was detected only at 4 wt.% comparing PCL/β-TCP/NaHCO_3_ 60/36/4 to PCL/β-TCP 60/40 at 1 and 2 days, losing statistical significance at the third day. Interestingly, an initially opposite effect of acidification was observed at 24 h for PCL/β-TCP/NaHCO_3_ 80/18/2 vs. PCL/β-TCP 80/20 (*p* < 0.05), which faded subsequently. This may be due to the lower presence of sodium bicarbonate (2 wt.%) and to the reduction in the surface area available for ion exchange [37].

The amount of protein BSA adsorbed to the samples was higher than to neat PCL for all the composites, but it was statistically significant only for those not containing sodium bicarbonate, with PCL/β-TCP 80/20 reaching the highest value (~4 mg/mL). The effect of bicarbonate on early cell adhesion was instead evident, as the both the formulations with NaHCO_3_ embedded were improved compared to their counterparts, and PCL/β-TCP/NaHCO_3_ 80/18/2 even outperformed neat PCL in a statistically significant way. However, PCL/β-TCP 60/40 and PCL/β-TCP/NaHCO_3_ 60/36/4 had fewer adherent cells than neat PCL in a statistically significant way. This apparent inconsistency with the protein adsorption results finds a possible explanation in the fact that cell adhesion is highly dependent not only on the quantity but also on the conformation and surface interaction of the adsorbed proteins [36,38]. This pattern was also present in the cellular morphology assay, since PCL/β-TCP 60/40 and PCL/β-TCP/NaHCO_3_ 60/36/4 allowed the adherent cells to spread less than neat PCL and the other two compounds, which is clearly portrayed by the lower mean area and perimeter measured. Notably, also, sodium bicarbonate rescued, at least in part, this negative outcome, as evident from a comparison between PCL/β-TCP 60/40 and PCL/β-TCP/NaHCO_3_ 60/36/4. The decoupling between protein adsorption and cell spreading has been reported before by Sawyer et al. [39] and dos Santos et al. [40]. The former study observed that mesenchymal stem cells were not capable of spreading on HA coated with RGD, suggesting that the RGD domain alone was not sufficient to promote full cell spreading; while the latter paper reported a downward trend in cell spreading on β-TCP, despite its good affinity with fibronectin adsorption.

While on PCL/β-TCP 80/20 and PCL/β-TCP/NaHCO_3_ 80/18/2 cells grew slightly less than on neat PCL at day 7, albeit not in a statistically significant way, PCL/β-TCP 60/40 and PCL/β-TCP/NaHCO_3_ 60/36/4 showed a remarkably slower proliferation rate. As all the composites were nevertheless biocompatible, several hypotheses may be formulated. An osteogenic differentiation process—theoretically—could have occurred earlier on the composites with higher β-TCP load, consistent with previous studies linking a decreased proliferation rate with the start of cellular commitment [41,42,43]. This speculation is to be evaluated based on further experiments assessing the expression level of the main osteogenic genes such as RUNX-2, osterix, alkaline phosphatase, collagen type I, osteocalcin, etc. On the other hand, varying the filler content, even if it was not strictly biomimicking, such as alumina-toughened zirconia, was demonstrated to exert selective biological response, dependent on the cell type tested [22].

In summary, within the limits of the present study, it can be concluded that sodium bicarbonate may be favorably introduced, as it increased the polar component of the surface energy, alkalinized the composite with higher β-TCP content, and attenuated its early negative cell response. Further investigation is needed to expand the filler range and deepen the knowledge of the possible mechanisms underpinning its mechanical features and long-term biological behavior.

## Figures and Tables

**Table 1:** Compositions of formulations.

Sample	PCL [g]	β-TCP [g]	NaHCO_3_ [g]
PCL	5	/	/
PCL/β-TCP 80/20	4	1	/
PCL/β-TCP/NaHCO_3_ 80/18/2	4	0.9	0.1
PCL/β-TCP 60/40	3	2	/
PCL/β-TCP/NaHCO_3_ 60/36/4	3	1.8	0.2

**Table 2:** Standard parameters for the measure of contact angle.

Liquid	γ [mN/m]	γP [mN/m]	γD [mN/m]
Water	72.8	43.7	29.1
Diiodomethane	50	2.6	47.4

**Table 3:** Surface free energy.

Sample	Surface Energy: Total [mN/m]	Surface Energy: Polar [mN/m]	Surface Energy: Dispersive [mN/m]
PCL	42.97	2.14	40.83
PCL/β-TCP 80/20	38.55	2.12	36.43
PCL/β-TCP/NaHCO_3_ 80/18/2	42.10	3.27	38.84
PCL/β-TCP 60/40	45.11	0.92	44.19
PCL/β-TCP/NaHCO_3_ 60/36/4	47.52	3.72	43.79

## Data Availability

Data supporting reported results are stored in the computers and laptops of the Bone and Dental Bioengineering Laboratory.

## Abbreviations

The following abbreviations are used in this manuscript:

PCL	Poly-ε-caprolactone
β-TCP	β-Tricalcium phosphate
CaPs	Calcium phosphate-based ceramics
HA	Hydroxyapatite
SEM	Scanning electron microscope
dH2O	Double-distilled water
CH2I2	Diiodomethane
OWRK	Owens–Wendt–Rabel–Kaelbele
AC	Contact area
hC	Contact penetration depth
BSA	Bovine serum albumin
PBS	Phosphate-buffered saline
ASCs	Adipose stem cells ASC52hTert
FBS	Fetal bovine serum
BFE	Best fitting ellipse
RLUs	Relative light units
OCA	Contact angle
EiT	Young’s modulus
HV	Vickers hardness
SFE	Surface free energy

## References

[1] Sallent I., Capella-Monsonís H., Procter P., Bozo I.Y., Deev R.V., Zubov D., Vasyliev R., Perale G., Pertici G., Baker J. (2020). The Few Who Made It: Commercially and Clinically Successful Innovative Bone Grafts. Front. Bioeng. Biotechnol..

[2] Ho-Shui-Ling A., Bolander J., Rustom L.E., Johnson A.W., Luyten F.P., Picart C. (2018). Bone Regeneration Strategies: Engineered Scaffolds, Bioactive Molecules and Stem Cells Current Stage and Future Perspectives. Biomaterials.

[3] Morris M.T., Tarpada S.P., Cho W. (2018). Bone Graft Materials for Posterolateral Fusion Made Simple: A Systematic Review. Eur. Spine J..

[4] Haugen H.J., Lyngstadaas S.P., Rossi F., Perale G. (2019). Bone Grafts: Which Is the Ideal Biomaterial?. J. Clin. Periodontol..

[5] Buser Z., Brodke D.S., Youssef J.A., Meisel H.-J., Myhre S.L., Hashimoto R., Park J.-B., Tim Yoon S., Wang J.C. (2016). Synthetic Bone Graft versus Autograft or Allograft for Spinal Fusion: A Systematic Review. J. Neurosurg. Spine.

[6] Ginebra M.-P., Espanol M., Maazouz Y., Bergez V., Pastorino D. (2018). Bioceramics and Bone Healing. EFORT Open Rev..

[7] Samavedi S., Whittington A.R., Goldstein A.S. (2013). Calcium Phosphate Ceramics in Bone Tissue Engineering: A Review of Properties and Their Influence on Cell Behavior. Acta Biomater..

[8] Hou X., Zhang L., Zhou Z., Luo X., Wang T., Zhao X., Lu B., Chen F., Zheng L. (2022). Calcium Phosphate-Based Biomaterials for Bone Repair. J. Funct. Biomater..

[9] Bohner M., Santoni B.L.G., Döbelin N. (2020). β-Tricalcium Phosphate for Bone Substitution: Synthesis and Properties. Acta Biomater..

[10] Yuan H., Fernandes H., Habibovic P., De Boer J., Barradas A.M.C., De Ruiter A., Walsh W.R., Van Blitterswijk C.A., De Bruijn J.D. (2010). Osteoinductive Ceramics as a Synthetic Alternative to Autologous Bone Grafting. Proc. Natl. Acad. Sci. USA.

[11] Hernigou P., Dubory A., Pariat J., Potage D., Roubineau F., Jammal S., Flouzat Lachaniette C.H. (2017). Beta-Tricalcium Phosphate for Orthopedic Reconstructions as an Alternative to Autogenous Bone Graft. Morphologie.

[12] Brochu B.M., Sturm S.R., Kawase De Queiroz Goncalves J.A., Mirsky N.A., Sandino A.I., Panthaki K.Z., Panthaki K.Z., Nayak V.V., Daunert S., Witek L. (2024). Advances in Bioceramics for Bone Regeneration: A Narrative Review. Biomimetics.

[13] Gentile P., Chiono V., Carmagnola I., Hatton P. (2014). An Overview of Poly(Lactic-Co-Glycolic) Acid (PLGA)-Based Biomaterials for Bone Tissue Engineering. Int. J. Mol. Sci..

[14] Di Maro M., Faga M.G., Malucelli G., Mussano F.D., Genova T., Morsi R.E., Hamdy A., Duraccio D. (2020). Influence of Chitosan on the Mechanical and Biological Properties of HDPE for Biomedical Applications. Polym. Test..

[15] Nguyen D.D., Luo L.-J., Yang C.-J., Lai J.-Y. (2023). Highly Retina-Permeating and Long-Acting Resveratrol/Metformin Nanotherapeutics for Enhanced Treatment of Macular Degeneration. ACS Nano.

[16] Malikmammadov E., Tanir T.E., Kiziltay A., Hasirci V., Hasirci N. (2018). PCL and PCL-Based Materials in Biomedical Applications. J. Biomater. Sci. Polym. Ed..

[17] Schäler K., Achilles A., Bärenwald R., Hackel C., Saalwächter K. (2013). Dynamics in Crystallites of Poly(ε-Caprolactone) as Investigated by Solid-State NMR. Macromolecules.

[18] Gleadall A., Pan J., Kruft M.-A., Kellomäki M. (2014). Degradation Mechanisms of Bioresorbable Polyesters. Part 1. Effects of Random Scission, End Scission and Autocatalysis. Acta Biomater..

[19] Prasad A., Kandasubramanian B. (2019). Fused Deposition Processing Polycaprolactone of Composites for Biomedical Applications. Polym.-Plast. Technol. Mater..

[20] Neufurth M., Wang X., Wang S., Steffen R., Ackermann M., Haep N.D., Schröder H.C., Müller W.E.G. (2017). 3D Printing of Hybrid Biomaterials for Bone Tissue Engineering: Calcium-Polyphosphate Microparticles Encapsulated by Polycaprolactone. Acta Biomater..

[21] Burr D.B., Allen M.R. (2019). Basic and Applied Bone Biology.

[22] Pedraza R., Mosca Balma A., Roato I., Orrico C., Genova T., Baima G., Berta G.N., Giura A., Ribotta L., Duraccio D. (2024). Early Biological Response to Poly(ε-Caprolactone)/Alumina-Toughened Zirconia Composites Obtained by 3D Printing for Peri-Implant Application. Polymers.

[23] Stringer C., Wang T., Michaelos M., Pachitariu M. (2021). Cellpose: A Generalist Algorithm for Cellular Segmentation. Nat. Methods.

[24] Stringer C., Pachitariu M. (2025). Cellpose3: One-Click Image Restoration for Improved Cellular Segmentation. Nat. Methods.

[25] Dietrich C.F. (2017). Uncertainty, Calibration and Probability: The Statistics of Scientific and Industrial Measurement.

[26] Hile D.D., Sonis S.T., Doherty S.A., Tian X.Y., Zhang Q., Jee W.S.S., Trantolo D.J. (2005). Dimensional Stability of the Alveolar Ridge After Implantation of a Bioabsorbable Bone Graft Substitute: A Radiographic and Histomorphometric Study in Rats. J. Oral Implantol..

[27] Hesaraki S., Zamanian A., Moztarzadeh F. (2008). The Influence of the Acidic Component of the Gas-foaming Porogen Used in Preparing an Injectable Porous Calcium Phosphate Cement on Its Properties: Acetic Acid *versus* Citric Acid. J. Biomed. Mater. Res. Part B Appl. Biomater..

[28] Cimatti B., Santos M.A.D., Brassesco M.S., Okano L.T., Barboza W.M., Nogueira-Barbosa M.H., Engel E.E. (2018). Safety, Osseointegration, and Bone Ingrowth Analysis of PMMA—Based Porous Cement on Animal Metaphyseal Bone Defect Model. J. Biomed. Mater. Res. Part B Appl. Biomater..

[29] Taylor E.A., Donnelly E. (2020). Raman and Fourier Transform Infrared Imaging for Characterization of Bone Material Properties. Bone.

[30] Taylor E.A., Mileti C.J., Ganesan S., Kim J.H., Donnelly E. (2021). Measures of Bone Mineral Carbonate Content and Mineral Maturity/Crystallinity for FT-IR and Raman Spectroscopic Imaging Differentially Relate to Physical–Chemical Properties of Carbonate-Substituted Hydroxyapatite. Calcif. Tissue Int..

[31] Chuang K.-W., Liu Y.-C., Balaji R., Chiu Y.-C., Yu J., Liao Y.-C. (2022). Enhancing Stability of High-Concentration β-Tricalcium Phosphate Suspension for Biomedical Application. Materials.

[32] Puszka A., Sikora J.W. (2022). New Segmented Poly(Thiourethane-Urethane)s Based on Poly(ε-Caprolactone)Diol Soft Segment: Synthesis and Characterization. Materials.

[33] Gao J., Wu S., Rogers M.A. (2012). Harnessing Hansen Solubility Parameters to Predict Organogel Formation. J. Mater. Chem..

[34] Di Maro M., Pedraza R., Mosca Balma A., Gomez d’Ayala G., Poggetto G.D., Malucelli G., Roato I., Duraccio D., Mussano F., Faga M.G. (2024). Influence of Dry-Mixing and Solvent Casting Blending Techniques on the Mechanical and Biological Behavior of Novel Biocompatible Poly (ε-Caprolactone)/Alumina-Toughened Zirconia Scaffolds Obtained by 3D Printing. J. Compos. Sci..

[35] Maleki-Ghaleh H., Hossein Siadati M., Fallah A., Zarrabi A., Afghah F., Koc B., Dalir Abdolahinia E., Omidi Y., Barar J., Akbari-Fakhrabadi A. (2021). Effect of Zinc-Doped Hydroxyapatite/Graphene Nanocomposite on the Physicochemical Properties and Osteogenesis Differentiation of 3D-Printed Polycaprolactone Scaffolds for Bone Tissue Engineering. Chem. Eng. J..

[36] Juan P.-K., Fan F.-Y., Lin W.-C., Liao P.-B., Huang C.-F., Shen Y.-K., Ruslin M., Lee C.-H. (2021). Bioactivity and Bone Cell Formation with Poly-ε-Caprolactone/Bioceramic 3D Porous Scaffolds. Polymers.

[37] Lei Y., Rai B., Ho K.H., Teoh S.H. (2007). In Vitro Degradation of Novel Bioactive Polycaprolactone—20% Tricalcium Phosphate Composite Scaffolds for Bone Engineering. Mater. Sci. Eng. C.

[38] Ruoslahti E. (1996). RGD and Other Recognition Sequences for Integrins. Annu. Rev. Cell Dev. Biol..

[39] Sawyer A.A., Hennessy K.M., Bellis S.L. (2005). Regulation of Mesenchymal Stem Cell Attachment and Spreading on Hydroxyapatite by RGD Peptides and Adsorbed Serum Proteins. Biomaterials.

[40] Dos Santos E.A., Farina M., Soares G.A., Anselme K. (2008). Surface Energy of Hydroxyapatite and β-Tricalcium Phosphate Ceramics Driving Serum Protein Adsorption and Osteoblast Adhesion. J. Mater. Sci. Mater. Med..

[41] Erisken C., Kalyon D.M., Wang H. (2008). Functionally Graded Electrospun Polycaprolactone and β-Tricalcium Phosphate Nanocomposites for Tissue Engineering Applications. Biomaterials.

[42] Owen T.A., Aronow M., Shalhoub V., Barone L.M., Wilming L., Tassinari M.S., Kennedy M.B., Pockwinse S., Lian J.B., Stein G.S. (1990). Progressive Development of the Rat Osteoblast Phenotype in Vitro: Reciprocal Relationships in Expression of Genes Associated with Osteoblast Proliferation and Differentiation during Formation of the Bone Extracellular Matrix. J. Cell. Physiol..

[43] Park S.H., Park S.A., Kang Y.G., Shin J.W., Park Y.S., Gu S.R., Wu Y.R., Wei J., Shin J.-W. (2017). PCL/β-TCP Composite Scaffolds Exhibit Positive Osteogenic Differentiation with Mechanical Stimulation. Tissue Eng. Regen. Med..

